# Genetically predicted alterations in thyroid function are associated with the risk of benign prostatic disease

**DOI:** 10.3389/fendo.2023.1163586

**Published:** 2023-04-17

**Authors:** Yan Huang, Cheng Chen, Wanqing Zhou, Qian Zhang, Yanfei Zhao, Dehao He, Zhi Ye, Pingping Xia

**Affiliations:** ^1^ Department of Anesthesiology, Xiangya Hospital of Central South University, Changsha, Hunan, China; ^2^ National Clinical Research Center for Geriatric Disorders, Central South University, Changsha, Hunan, China

**Keywords:** thyroid function, benign prostatic diseases, Mendelian randomization, genome-wide association study, causality

## Abstract

**Background:**

Benign prostatic diseases (BPDs), such as benign prostate hyperplasia (BPH) and prostatitis, harm the quality of life of affected patients. However, observational studies exploring the association between thyroid function and BPDs have hitherto yielded inconsistent results. In this study, we explored whether there is a causal genetic association between them using Mendelian randomization (MR) analysis.

**Methods:**

We used publicly available summary statistics from the Thyroidomics Consortium and 23andMe on thyrotropin (TSH; 54,288 participants), thyroxine [free tetraiodothyronine (FT4); 49,269 participants], subclinical hypothyroidism (3,440 cases and 49,983 controls), overt hypothyroidism (8,000 cases and 117,000 controls), and subclinical hyperthyroidism (1,840 cases and 49,983 controls) to screen for instrumental variables of thyroid function. Results for BPD such as prostatic hyperplasia (13,118 cases and 72,799 controls) and prostatitis (1,859 cases and 72,799 controls) were obtained from the FinnGen study. The causal relationship between thyroid function and BPD was primarily assessed using MR with an inverse variance weighted approach. In addition, sensitivity analyses were performed to test the robustness of the results.

**Results:**

We found that TSH [OR (95% CI) = 0.912(0.845-0.984), *p* =1.8 x 10^-2^], subclinical hypothyroidism [OR (95% CI) = 0.864(0.810-0.922), *p* =1.04 x 10^-5^], and overt hypothyroidism [OR (95% CI) = 0.885 (0.831-0. 944), *p* =2 x 10^-4^] had a significant effect on genetic susceptibility to BPH, unlike hyperthyroidism [OR (95% CI) = 1.049(0.990-1.111), *p* =1.05 x 10^-1^] and FT4 [OR (95% CI) = 0.979(0.857-1.119), *p* = 7.59 x 10^-1^] had no effect. We also found that TSH [OR (95% CI) =0.823(0.700-0.967), *p* = 1.8 x 10^-2^] and overt hypothyroidism [OR (95% CI) = 0.853(0.730-0.997), *p* = 4.6 x 10^-2^] significantly influenced the prostatitis, whereas FT4 levels [OR (95% CI) = 1.141(0.901-1.444), *p* = 2.75 x 10^-1^], subclinical hypothyroidism [OR (95% CI) =0. 897(0.784- 1.026), *p* = 1.12 x 10^-1^], and hyperthyroidism [OR (95% CI) = 1.069(0.947-1.206), *p* = 2.79 x 10^-1^] did not have a significant effect.

**Conclusion:**

Overall, our study results suggest that hypothyroidism and TSH levels influence the risk of genetically predicted BPH and prostatitis, providing new insights into the causal relationship between thyroid function and BPD.

## Introduction

Benign prostatic diseases (BPDs), such as benign prostatic hyperplasia (BPH) and prostatitis, significantly impact men’s quality of life. It is now understood that BPH may lead to the development of prostate cancer (1). However, the etiology of BPH remains unclear. The possible influencing factors are inflammation, bacterial infection, and endocrine hormones (2). From an endocrinological perspective, several studies (3, 4) have been performed to investigate the unique role of endocrine hormones, including testosterone, estrogen, vitamin D, and sex hormone–binding protein. However, the relationship between thyroid function and BPH has been largely understudied.

Thyroid hormones (THs) are extensively involved in cell growth, metabolism, and differentiation. Current evidence suggests that THs activate the TH response element (TRE) in the promoter of TH target genes by binding to nuclear TH receptors (THRs) (5). The thyroid-stimulating hormone (TSH) is secreted by the pituitary gland, promotes the secretion of THs, and is regulated by THs’ feedback control. Hyperthyroidism and hypothyroidism are the most direct manifestations of dysregulated TH secretion. It has been reported that THRs are mainly present in the epithelial cells of the prostate (6), suggesting that nuclear THRs can regulate glandular activity. In the most extensive observational study to date (5,708 Korean men), prostate volume was associated with FT4 but not with TSH (7). However, the data were taken from a single institution cross-sectional study and may have some limitations in causal inference. BPH and urinary retention have been associated with a poor prognosis in prostatitis (8). It is well established that patients with prostatitis are more susceptible to BPH (9), suggesting a possible relationship between prostate enlargement and prostatitis. Furthermore, the mechanisms underlying prostate enlargement may participate in the pathogenesis of prostatitis. However, no relevant studies have demonstrated a direct association between thyroid hormones and the risk of prostatitis. In addition, traditional observational studies may be subject to inherent confounding or selection bias, which prompted us to use new methods to explore the true picture.

We hypothesized that thyroid function (THs and thyroid disease) is causally related to the occurrence of BPD (BPH and prostatitis) in the general population, and Mendelian randomization (MR) analysis could be used to address these unmet research needs. In MR, genetic variation is used as an instrumental variable to estimate the causal effect of the exposure (thyroid function) on the outcome (BPD). Due to the random assignment of variance, MR is not susceptible to confounding, measurement error, and reverse causality (10). The present study used a two-sample MR approach to investigate whether thyrotropin (TSH), thyroxine [free tetraiodothyronine (FT4)], hypothyroidism (subclinical and overt), and hyperthyroidism are associated with BPH and prostatitis using publicly available summary-level data from the Genome-Wide Association Study (GWAS).

## Methods

### Data source and study population

Our study was conducted using publicly available summary-level GWAS data. The design of this study was informed by the Strengthening the Reporting of Observational Studies in Epidemiology Using Mendelian Randomization (STROBE-MR) checklist (11). Detailed information on study characteristics, participants, and ethical statements for each dataset were extracted from the original publication or website. We selected TSH, FT4, subclinical hypothyroidism, and subclinical hyperthyroidism–related GWAS from the Thyroidomics Consortium (12, 13), all within the cohort-specific reference range and without significant thyroid disease (thyroid surgery or medication use). Overt hypothyroidism–related GWAS cases from 23andMe included subclinical and overt hypothyroidism. The GWAS associated with BPD was obtained from FinnGen. The FinnGen study is a unique study that combines genomic information with digital healthcare data from participants aged 18 years and older living in Finland (14). All participants were of European ancestry, and there was no overlap between the exposure (thyroid function) and outcome (BPH) samples. Single-nucleotide polymorphism (SNP) locations are based on Genome Reference Consortium Human Build 37 (GRCh37). All detailed descriptions are included in the table (**Supplementary Table S1**).

### Genetic variants

**Figure 1:**
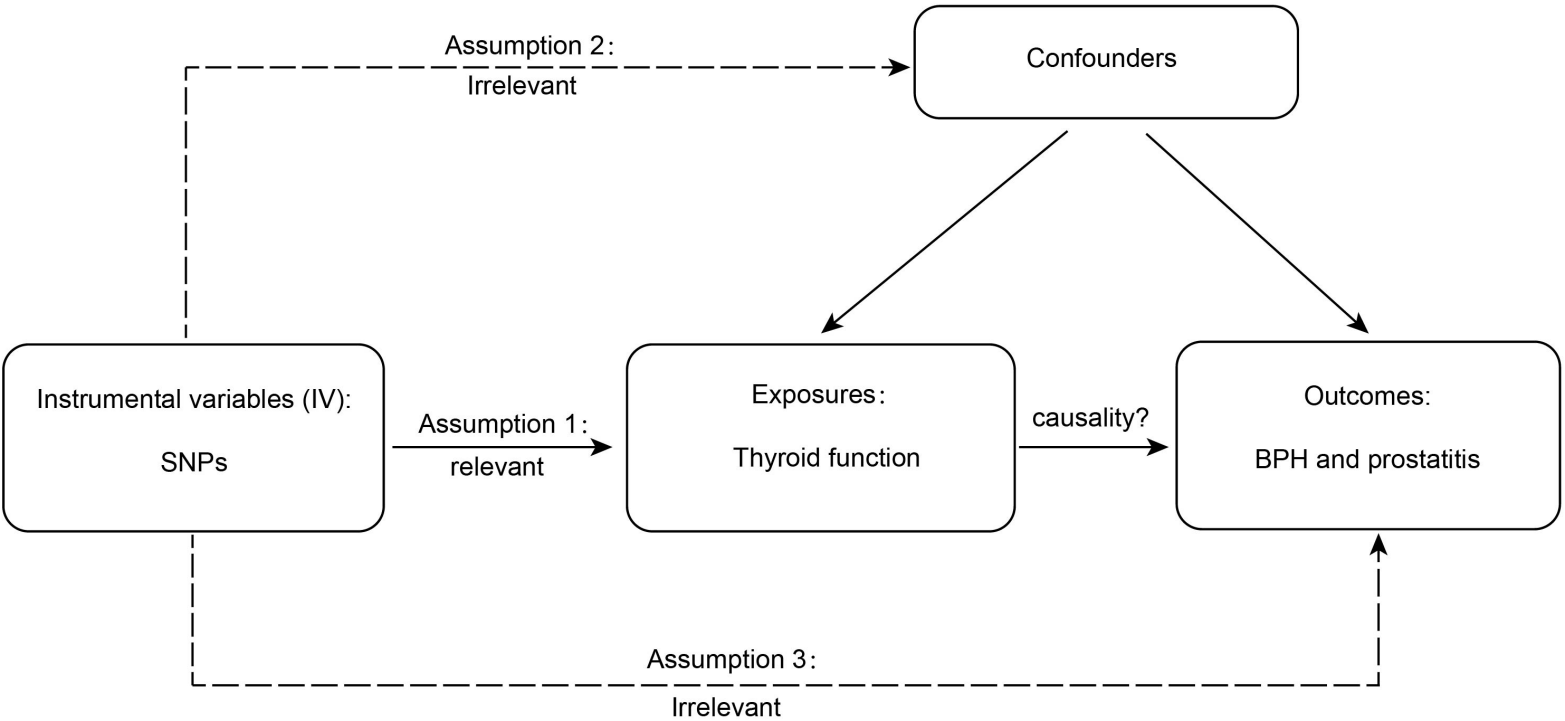
The detailed principles of Mendelian randomization study. BPH, benign prostatic hyperplasia.

Valid MR is based on three assumptions (15), and the detailed principles are shown in **Figure 1**. First, genetic variation is significantly associated with thyroid function; second, as an instrumental variable (IV) for exposure, the data should not be associated with the confounders of thyroid function and BPD; and, finally, BPD should only be influenced by genetic variation through thyroid function. SNPs are commonly used as independent genetic predictors and are IVs if they meet stringent assumptions (16). For quality control, we screened SNPs with a genome-wide significance threshold (*p* < 5 × 10^-8^). SNPs with linkage dependence and excessively short genetic distance (r^2^ < 1 × 10^-3^ and kb > 1 − 10^4^) were excluded. We also removed variants with F values <10 as an indicator of IV strength. SNPs with mismatching alleles were also removed to reduce potential bias from weak instrumentation (10).

### Mendelian randomization analysis

We used a two-sample MR approach to determine the association between thyroid function and BPD. The effect of exposure on the outcome can be estimated from the ratio of the genetic outcome and genetic exposure association estimates. In addition, if the genetic variants are not in linkage disequilibrium, the ratio estimates for each genetic variant can be combined into an overall estimate using a formula from the literature known as inverse variance weighted (IVW) methods (17). When all SNPs met the requirement for valid instrumental variables, causality was mainly assessed by IVW methods. Furthermore, MR–Egger regression, weighted median, weighted mode, and simple mode can provide complementary effects for additional evaluation in the presence of outliers (18). If there were no weak IV, we used IVW as the primary outcome and the other methods as secondary outcomes (19). If the Mendelian Randomization Pleiotropy RESidual Sum and Outlier (MR-PRESSO) method detected significant horizontal pleiotropy, we removed outlier variants and repeated the MR analysis (20). We used the leave-one-out test to identify the effect of individual SNP effects (21). Cochran’s Q test was used to calculate heterogeneity, and *p* < 0.05 indicated the presence of considerable heterogeneity and the need to exclude SNPs (21). The MR–Egger method provided the estimates of horizontal multiplicity from the intercepts of linear regressions of SNP-outcome and SNP-exposure association estimates (22). *p* < 0.05 indicated the presence of nominal significance (23). We used TwoSampleMR 0.5.6 and the MR-PRESSO 1.0 package in R version 4.2.1 for the analysis.

## Results

We found that 59 SNPs, 31 SNPs, 8 SNPs, 18 SNPs, and 7 SNPs from the Thyroidomics Consortium with 23andMe were associated with TSH, FT4, subclinical hypothyroidism, overt hypothyroidism, and subclinical hyperthyroidism, respectively. Due to the concordance of the outcomes, some SNPs were eliminated after harmonization. All F-statistics reached the threshold value (F > 10) (**Supplementary Table S2**). The detailed data for all five methods we used are provided in the Supplementary Material (**Supplementary Table 3**).

### Thyroid hormones and benign prostatic diseases

**Figure 2:**
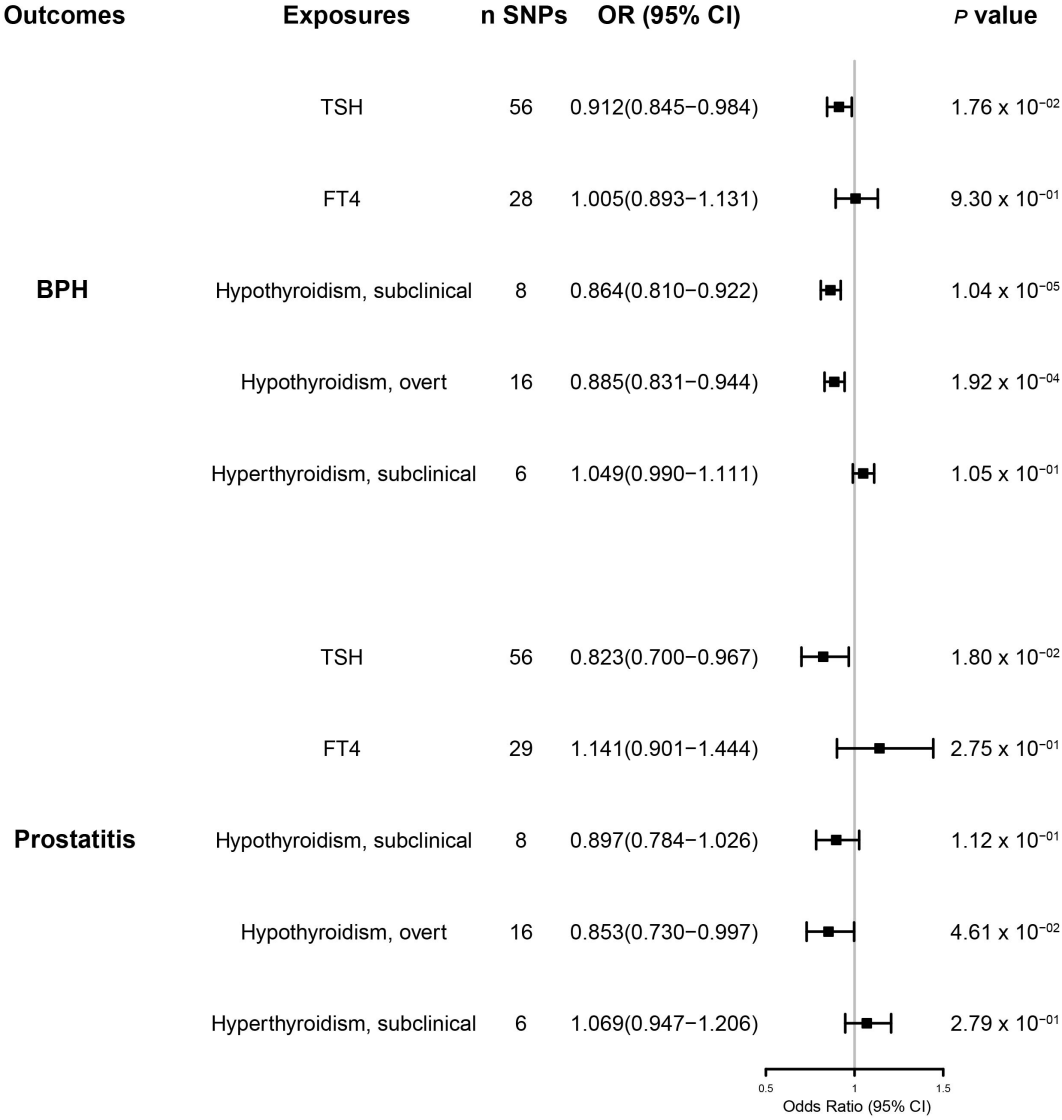
The results of the inverse variance weighted method to assess the causal role of thyroid function in the risk of benign prostatic hyperplasia and prostatitis. BPH, benign prostatic hyperplasia; CI, confidence interval; SNPs, single-nucleotide polymorphisms.

Our MR-IVW analysis suggested a causal relationship between thyroid hormones and BPD (**Figure 2** and **Supplementary Figures 1, 2**). Genetically predicted elevated TSH levels were potentially associated with a reduced risk of BPH [OR (95% CI) = 0.912(0.845–0.984); *p* = 1.8 × 10^-2^] and prostatitis [OR (95% CI) = 0.823 (0.700–0.967); *p* = 1.8 × 10^-2^]. However, genetically predicted FT4 levels were not significantly associated with either BPH or prostatitis risk [BPH OR (95% CI) = 0.979 (0.857–1.119), *p* = 7.59 × 10^-1^; prostatitis OR (95% CI) =1.141(0.901–1.444), *p* = 2.75 × 10^-1^)].

No significant heterogeneity was found between TSH and BPD during Cochran’s Q test (*p* > 0.05) (**Table 1**). The MR–Egger test [BPH (*p* = 0.762); prostatitis (*p* = 0.608)] or the MR-PRESSO global test [BPH (*p* = 0.605); prostatitis (*p* = 0.625)] showed no pleiotropy (**Table 2**). The sensitivity analysis of FT4 and BPH indicated the presence of aberrant SNPs. After excluding rs4149056 by the MR-PRESSO test, no heterogeneity or pleiotropy was found (**Table 2**). There was no heterogeneity between FT4 and prostatitis (*p* > 0.05) (**Table 1**). The MR–Egger test (*p* = 0.338) or the MR-PRESSO global test (*p* = 0.468) showed no pleiotropy (**Table 2**). No single genetic variant that strongly drove the overall effect of TSH or FT4 on BPD was identified in the leave-one-out analysis (**Supplementary Figures 3, 4**).

**Table 1:** Heterogeneity tests in the causality of thyroid function and benign prostatic diseases.

	Benign prostate hyperplasia				Prostatitis			
	Method	Q	df	*p* value	Method	Q	df	*p* value
TSH	IVW	51.895	55	0.594	IVW	51.150	55	0.622
	MR–Egger	51.802	54	0.560	MR–Egger	50.884	54	0.595
FT4 (before correction)	IVW	42.169	28	0.042	IVW	29.227	28	0.401
	MR–Egger	42.131	27	0.032	MR–Egger	28.233	27	0.399
FT4 (after correction)	IVW	31.202	27	0.263	–	–	–	–
	MR–Egger	31.135	26	0.223	–	–	–	–
Hypothyroidism, overt	IVW	12.591	15	0.634	IVW	19.832	15	0.178
	MR–Egger	10.768	14	0.704	MR–Egger	19.803	14	0.136
Hypothyroidism, subclinical	IVW	7.343	7	0.394	IVW	2.374	7	0.936
	MR–Egger	7.303	6	0.294	MR–Egger	2.292	6	0.891
Hyperthyroidism, subclinical	IVW	5.094	5	0.405	IVW	2.221	5	0.818
	MR–Egger	4.320	4	0.364	MR–Egger	1.629	4	0.804

Q, Cochran’s Q statistic; df, degrees of freedom; IVW, inverse variance weighted.

**Table 2:** Pleiotropy test in the causality of thyroid function and benign prostatic diseases.

	Benign prostate hyperplasia					Prostatitis				
	MR–Egger			MR-PRESSO		MR–Egger			MR-PRESSO	
	Egger Intercept	SE	*p *value	Global test	*p* value	Egger Intercept	SE	*p *value	Global test	*p *value
TSH	0.002	0.006	0.762	53.739	0.605	-0.007	0.014	0.608	53.16	0.625
FT4 (before correction)	-0.002	0.011	0.877	50.526	0.026	-0.018	0.018	0.338	32.435	0.468
FT4 (after correction)	0.002	0.009	0.814	38.765	0.167	–	–	–	–	–
Hypothyroidism, subclinical	-0.005	0.028	0.862	9.409	0.471	-0.015	0.054	0.784	3.094	0.943
Hypothyroidism, overt	0.014	0.011	0.198	16.789	0.554	-0.004	0.027	0.888	22.376	0.252
Hyperthyroidism, subclinical	0.031	0.037	0.445	6.934	0.471	0.058	0.075	0.485	3.992	0.772

### Hypothyroidism and benign prostatic diseases

Subclinical and overt hypothyroidism predicted primarily by the IVW method was associated with a reduced risk of BPH [subclinical hypothyroidism OR (95% CI) = 0.864(0.810–0.922), *p* = 1.04 × 10^-5^; overt hypothyroidism OR (95% CI) = 0.885(0.831–0.944), *p* = 2 ×10^-4^)] (**Figure 2** and **Supplementary Figure 1**). Genetically predicted overt hypothyroidism was associated with a reduced risk of prostatitis [OR (95% CI) = 0.853(0.730–0.997), *p* = 4.6 × 10^-2^)], whereas subclinical hypothyroidism was not related to prostatitis [OR (95% CI) = 0.897(0.784–1.026)], *p* = 1.12 ×10^-1^)] (**Figure 2** and **Supplementary Figure 2**).

For BPH, Cochran’s Q test showed no significant heterogeneity (p > 0.05) for hypothyroidism (subclinical and overt) (**Table 1**). The MR–Egger test [subclinical hypothyroidism (intercept = -0.005, *p* = 0.862); overt hypothyroidism (*p* = 0.198)] or the MR-PRESSO global test [subclinical hypothyroidism (*p* = 0.471); overt hypothyroidism (*p* = 0.554)] showed no pleiotropy (**Table 2**). Leave-one-out analysis revealed no specific genetic variation (**Supplementary Figure 3**). For prostatitis, Cochran’s Q test and leave-one-out analysis both demonstrated the robustness of this association (*p* > 0.05) (**Table 1** and **Supplementary Figure 4**). The MR–Egger test [subclinical hypothyroidism (*p* = 0.784); overt hypothyroidism (*p* = 0.888)] or the MR-PRESSO global test [subclinical hypothyroidism (*p* = 0.943); overt hypothyroidism (*p* = 0.252)] indicated low pleiotropy (**Table 2**).

### Hyperthyroidism and benign prostatic diseases

Genetic susceptibility to subclinical hyperthyroidism was not correlated with the risk of BPH and prostatitis, according to the IVW results [BPH OR (95% CI) = 1.049(0.990–1.111), *p* =1.05 × 10^-1^; prostatitis OR (95% CI) = 1.069(0.947–1.206), *p* =2.79 × 10^-1^)] (**Figure 2** and **Supplementary Figures 1, 2**). Cochran’s Q test showed no significant heterogeneity (*p* > 0.05) (**Table 1**). The MR–Egger test [BPH (*p* = 0.888); prostatitis (*p* = 0.485)] or MR-PRESSO global test [BPH (*p* = 0.252); prostatitis (*p* = 0.772)] did not indicate any evidence of pleiotropy (**Table 2**). In addition, the leave-one-out test suggests that there is no overwhelming IV interference on the reliability of the results (**Supplementary Figures 3, 4**).

## Discussion

The present study investigated the relationship between thyroid function and BPH. We found that TSH levels and the occurrence of subclinical and overt hypothyroidism were potentially associated with the risk of developing BPH. TSH and overt hypothyroidism significantly affected the risk of prostatitis. Interestingly, FT4 levels and the occurrence of subclinical hyperthyroidism did not have a significant effect on BPH.

Although the relationship between TSH or FT4 and BPH has been studied, the exact association remains unclear. In an observational study of 40 patients with confirmed BPH and 40 age-matched non-patients, TSH was negatively associated with prostate size (24). However, no association was observed in another study (7). A positive correlation was observed between prostate volume and elevated FT4 levels among patients with documented BPH (n = 5,708) (7). A small case–control study (40 cases *vs*. 40 controls) found a significant increase in serum FT3 and FT4 in patients with BPH (24). However, another prospective study reported no association between the thyroid status (total T4 or FT4) and BPH risk (25). There is an association between BPH and prostatitis (26, 27). Therefore, although no direct relationship between TSH or FT4 and prostatitis has been reported, we can seek an explanation from BPH-related studies. The most common cause of prostatitis is the reflux of infected urine into the prostatic ducts (episodic urethral infection), and the incidence of prostatic hyperplasia is associated with the chance of reflux, thus influencing the risk of prostatitis (8). In the data from those who underwent transurethral resection of the prostate (TURP) (n = 374), 70% of patients with urinary retention and 45% of patients with lower urinary tract symptoms (LUTS) had acute and/or chronic intraprostatic inflammation (ACI) (28). Another study revealed that among patients with self-reported prostatitis, 57% had a history of BPH (29). These findings inspired us to re-examine TSH and FT4 in prostatitis.

Chronic inflammation reportedly plays an important role in the pathogenesis and progression of BPH. It may lead to altered growth patterns of prostate stromal cells (PSCs) and fibromuscular tissue in BPH, resulting in wound-healing-like changes that drive local growth factor production and angiogenesis in the tissue (30, 31). It has been found that, in BPH specimens with a low inflammatory response, the immune response is predominantly type 1, whereas, in nodular BPH with chronic inflammatory infiltration, it is mainly type 0 or 2 (32). In the Reduction by Dutasteride of Prostate Cancer Events trial, among 4,109 men receiving a placebo (non-androgen), men with chronic prostate inflammation at baseline had a more significant increase in prostate volume (33). This suggests that BPH and prostatitis may be interchangeable. It has been established that THs directly affect inflammation. Several studies have shown that THs mediate many inflammatory pathways, including regulating xanthine oxidase production, *via* the Toll-like receptors 4/nuclear factor kappa beta (TLR4/ NF-κβ) pathway (34) and controlling Interleukin 6 (IL-6) signaling during toxemia (35). THs attach to the integrin αvβ3 and reactive the PI3K-AKT signaling pathway, generating large amounts of eactive oxygen species (ROS) and triggering the release of pro-inflammatory cytokines from the NLR Family Pyrin Domain Containing 3 (NLRP3) complex (36). THs have been shown to increase prostate cell proliferation and the activation of inflammatory signaling in primary fibroblasts from human BPH samples (37). The above studies overlap in their assertion that inflammation and oxidative stress represent potential mechanisms by which thyroid hormones act in BPD.

Interestingly, our study revealed an inconsistent association between genetically predicted TSH and FT4 in BPH. One possible explanation for this inconsistency is that biologically active T3 acts directly on receptors in the prostate. Although FT4 represents an accurate indicator of thyroid function, it may not reflect localized T3 levels acting on the prostate. Current evidence suggests that the binding of T3 mediates the direct classical effect of THs on nuclear receptors (38). Landmark studies in adult rats with normal thyroid function have shown that approximately 20% of prostate T3 content is derived from T4 deiodination, with the rest derived from native plasma T3 (39). Some observational studies have shown increased T3 levels (40) and no change in T4 levels in patients with BPH. Another possible mechanism is that the direct action of thyroid hormones on the prostate receptors may not be predominant and other non-direct actions may be more critical. A seminal study showed that physiological T4 supplementation during the postnatal period (days 1–35) decreased prostate weight in prepubertal rats (41), whereas an increase in prostate weight was observed in T4-treated castrated rats (42). Although these two early studies did not measure TSH, FT4, and T3 levels, preventing us from determining the relationship between the thyroid functional status and prostate volume, their findings suggest the existence of a thyroid–sex hormone–prostate axis. We speculate that this indirect action may act through TSH released by the pituitary gland, which may determine prostate function through sex hormone levels. A recent MR study showed that TSH, but not FT4 levels, correlated with total serum testosterone concentrations (12). Interestingly, it has been shown that androgens stimulate prostate epithelial and mesenchymal cell differentiation and proliferation (43). Epidemiological studies have shown that circulating serum levels of endogenous sex hormones correlate with prostate volume and may influence the natural course of BPH (44). Elevated TSH levels and symptomatic improvement have been observed in patients with BPH treated with testosterone inhibitors (43). Given the previously mentioned, our findings do not exclude an association between thyroid hormones and BPH.

The relationship between hypothyroidism and the prostate has been reported previously in related studies. Hypothyroidism reduces luteinizing hormone and testosterone levels in adult rats, as well as indirectly reducing prostate weight (45). A prospective analysis (326 cases/9,981 participants) revealed that subclinical and overt hypothyroidism were not linked to an increased risk of prostate cancer (46). In contrast, although confounding cannot be completely ruled out, a decreased risk was reported in smoking men with overt hypothyroidism (20 cases/total 75) (46), suggesting that hypothyroidism may be associated with prostate cell proliferation. Subclinical and overt hypothyroidism were both associated with a reduced risk of BPH, further strengthening the credibility of the present study results. However, for prostatitis, only overt hypothyroidism was significantly associated, which may be attributed to the fact that patients with overt hypothyroidism have significantly higher TSH levels than patients with subclinical hypothyroidism, exerting a greater impact on prostate volume and thus on prostatitis (47).

A retrospective study demonstrated that hyperthyroidism significantly increased the odds of BPH (191 cases/832 patients), but the association was no longer significant when the data were corrected for age and other metabolic diseases (48). However, studies have also shown an association between hyperthyroidism and BPH (7). In the studies of prostate cancer, hyperthyroidism was significantly associated with increased cancer risk (46, 49). In contrast, this association was not significant in other studies (50–52), revealing a complex hyperthyroid-prostate cell proliferation profile. From a genetic correlation perspective, our study confirms the absence of a causal relationship between subclinical hyperthyroidism and BPH. However, this association cannot be excluded because the subjects were from a single cohort and had a single phenotype.

A strength of our study is the MR design that could simulate a randomized controlled trial. Our instrumental variable SNP was randomly assigned at conception, avoiding confounding bias. MR also avoids reverse causal effects compared with other observational studies. Moreover, large sample-based population studies on the relationship between thyroid function and prostatitis have yet to be reported. Indeed, our finding of an inconsistent association between the severity of hypothyroidism and prostatitis may inspire future studies. The limitations of the present study should be acknowledged. First, all GWAS data come from people of European ancestry, and whether our findings can be generalized to other populations remains to be investigated. In addition, sample sets from Europe may be shared, which could lead to the overuse of genetic data. Furthermore, since we only examined the effect of the subclinical phenotype on hyperthyroidism, our findings do not necessarily extend to more severe conditions. Therefore, we cannot exclude that overt hyperthyroidism is associated with BPD.

## Conclusion

Overall, the present study substantiated that elevated TSH levels and the development of hypothyroidism reduce the risk of prostate enlargement and prostatitis, suggesting an association between thyroid disease and BPH at the endocrine level. Our findings suggest that TSH may be a better predictor of BPH than FT4 levels and that the euthyroid status may have a preventive and therapeutic effect on BPH.

## Data availability statement

The original contributions presented in the study are included in the article/**Supplementary Material**. Further inquiries can be directed to the corresponding author.

## Ethics statement

All summary level data are from de-identified genome-wide association studies (GWAS) and are available for download. These studies were conducted in accordance with the Declaration of Helsinki and approved by the relevant institutional ethics committees.

## Author contributions

PX and YH conceived the research and determined the structure and layout of the paper. YH, CC, WZ, QZ, and DH collected and interpreted the data. CC, WZ, and YZ helped to analyze the results of this Mendelian randomization study. ZY and PX contributed to revising and finalizing the article. All authors contributed to the article and approved the submitted version.
