# Supplementary material for: Genetically predicted alterations in thyroid function are associated with the risk of benign prostatic disease
Source: Front Endocrinol (Lausanne). 2023 Apr 17;14:1163586. doi: 10.3389/fendo.2023.1163586 (PMC10153094; doi:10.3389/fendo.2023.1163586)
Supplement: Supplementary file 1 [file DataSheet_1.pdf]

Supplementary Material

Genetically Predicted Alterations in Thyroid Function are Associated with the Risk of Benign Prostatic Disease

Yan Huang, Cheng Chen, Wanqing Zhou, Qian Zhang, Yanfei Zhao, Dehao He, Zhi Ye, Pingping Xia*

*** Correspondence:** Pingping Xia: 310132585@qq.com

1 Supplementary Figures and Tables

1.1 Supplementary Figures

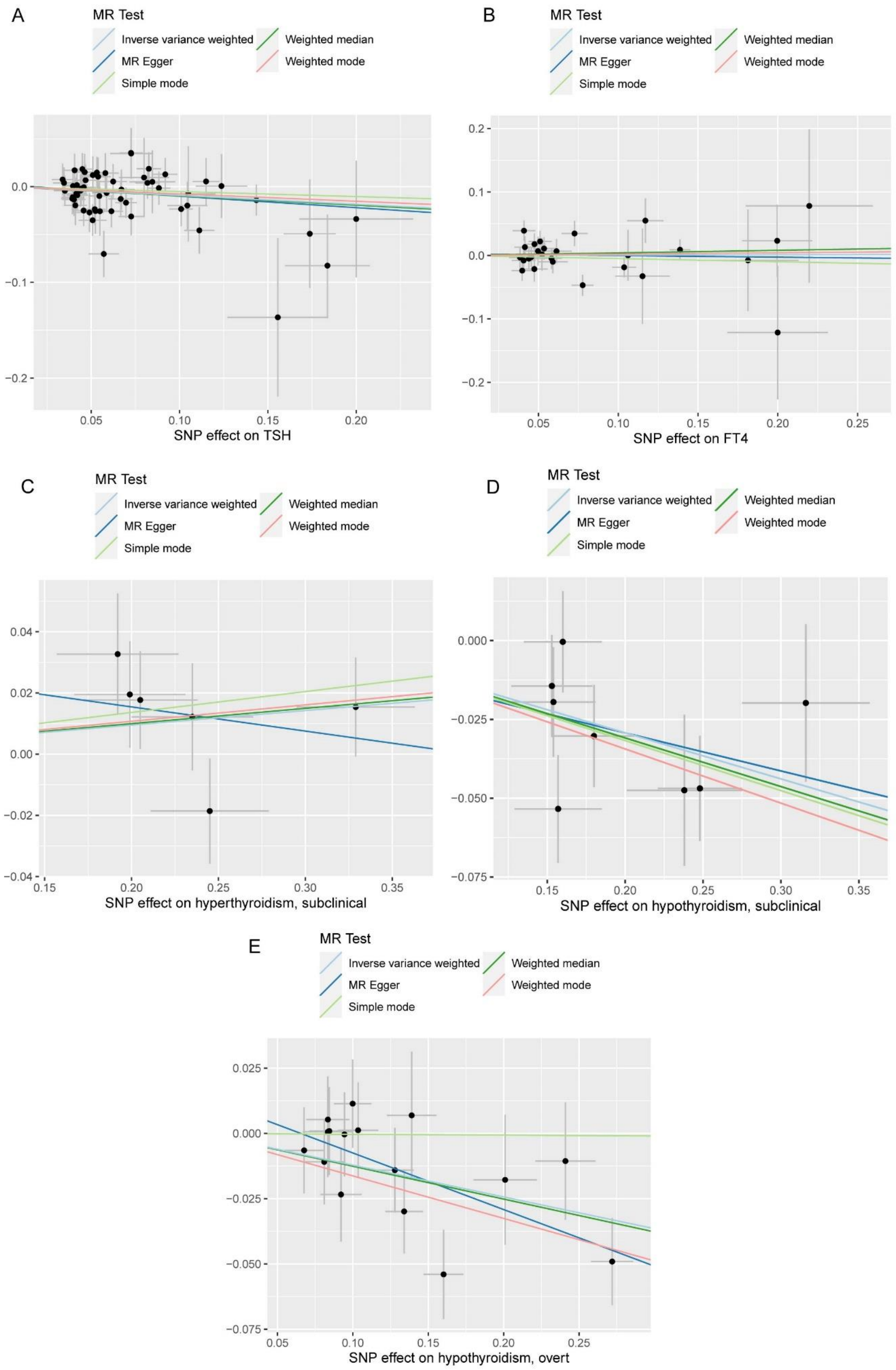
A
MR Test
Inverse variance weighted
MR Egger
Simple mode
Weighted median
Weighted mode
0.0
−0.1
−0.2
0.05
0.10
0.15
0.20
SNP effect on TSH
B
MR Test
Inverse variance weighted
MR Egger
Simple mode
Weighted median
Weighted mode
0.2
0.1
0.0
−0.1
−0.2
0.05
0.10
0.15
0.20
0.25
SNP effect on FT4
C
MR Test
Inverse variance weighted
MR Egger
Simple mode
Weighted median
Weighted mode
0.04
0.02
0.00
−0.02
−0.04
0.15
0.20
0.25
0.30
0.35
SNP effect on hyperthyroidism, subclinical
D
MR Test
Inverse variance weighted
MR Egger
Simple mode
Weighted median
Weighted mode
0.000
−0.025
−0.050
−0.075
0.15
0.20
0.25
0.30
0.35
SNP effect on hypothyroidism, subclinical
E
MR Test
Inverse variance weighted
MR Egger
Simple mode
Weighted median
Weighted mode
0.025
0.000
−0.025
−0.050
−0.075
0.05
0.10
0.15
0.20
0.25
SNP effect on hypothyroidism, overt

Supplementary Figure 1. Scatter plot of 5 methods for causal association between thyroid function and risk of BPH. Each black dot represents a SNP, plotted by SNP estimates in circulating (A) TSH, (B) FT4, (C) hyperthyroidism, subclinical, (D) hypothyroidism, overt, and (E) hypothyroidism, subclinical individuals at risk of BPH with standard error bars. The slope of the line corresponds to the causal estimates obtained by five different methods. SNPs, single nucleotide polymorphisms; MR, Mendelian randomization. BPH, benign prostate hyperplasia.

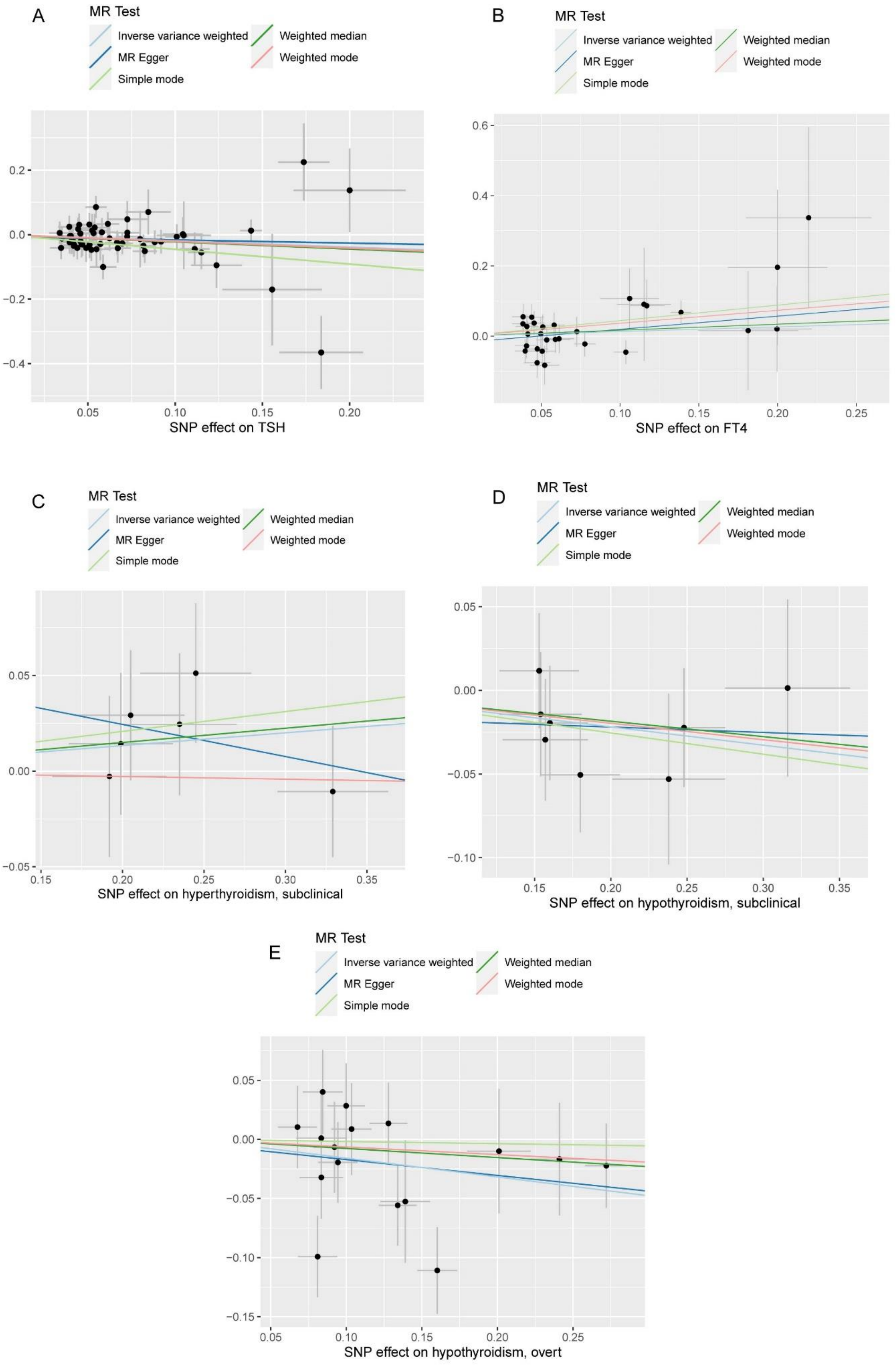
A
MR Test
Inverse variance weighted
Weighted median
MR Egger
Weighted mode
Simple mode
0.2
0.0
−0.2
−0.4
0.05
0.10
0.15
0.20
SNP effect on TSH
B
MR Test
Inverse variance weighted
Weighted median
MR Egger
Weighted mode
Simple mode
0.6
0.4
0.2
0.0
0.05
0.10
0.15
0.20
0.25
SNP effect on FT4
C
MR Test
Inverse variance weighted
Weighted median
MR Egger
Weighted mode
Simple mode
0.05
0.00
−0.05
0.15
0.20
0.25
0.30
0.35
SNP effect on hyperthyroidism, subclinical
D
MR Test
Inverse variance weighted
Weighted median
MR Egger
Weighted mode
Simple mode
0.05
0.00
−0.05
−0.10
0.15
0.20
0.25
0.30
0.35
SNP effect on hypothyroidism, subclinical
E
MR Test
Inverse variance weighted
Weighted median
MR Egger
Weighted mode
Simple mode
0.05
0.00
−0.05
−0.10
−0.15
0.05
0.10
0.15
0.20
0.25
SNP effect on hypothyroidism, overt

Supplementary Figure 2. Scatter plot of 5 methods for causal association between thyroid function and risk of prostatitis. Each black dot represents a SNP, plotted by SNP estimates in circulating (A) TSH, (B) FT4, (C) hyperthyroidism, subclinical, (D) hypothyroidism, overt, and (E) hypothyroidism, subclinical individuals at risk of prostatitis with standard error bars. The slope of the line corresponds to the causal estimates obtained by five different methods. SNPs, single nucleotide polymorphisms; MR, Mendelian randomization.

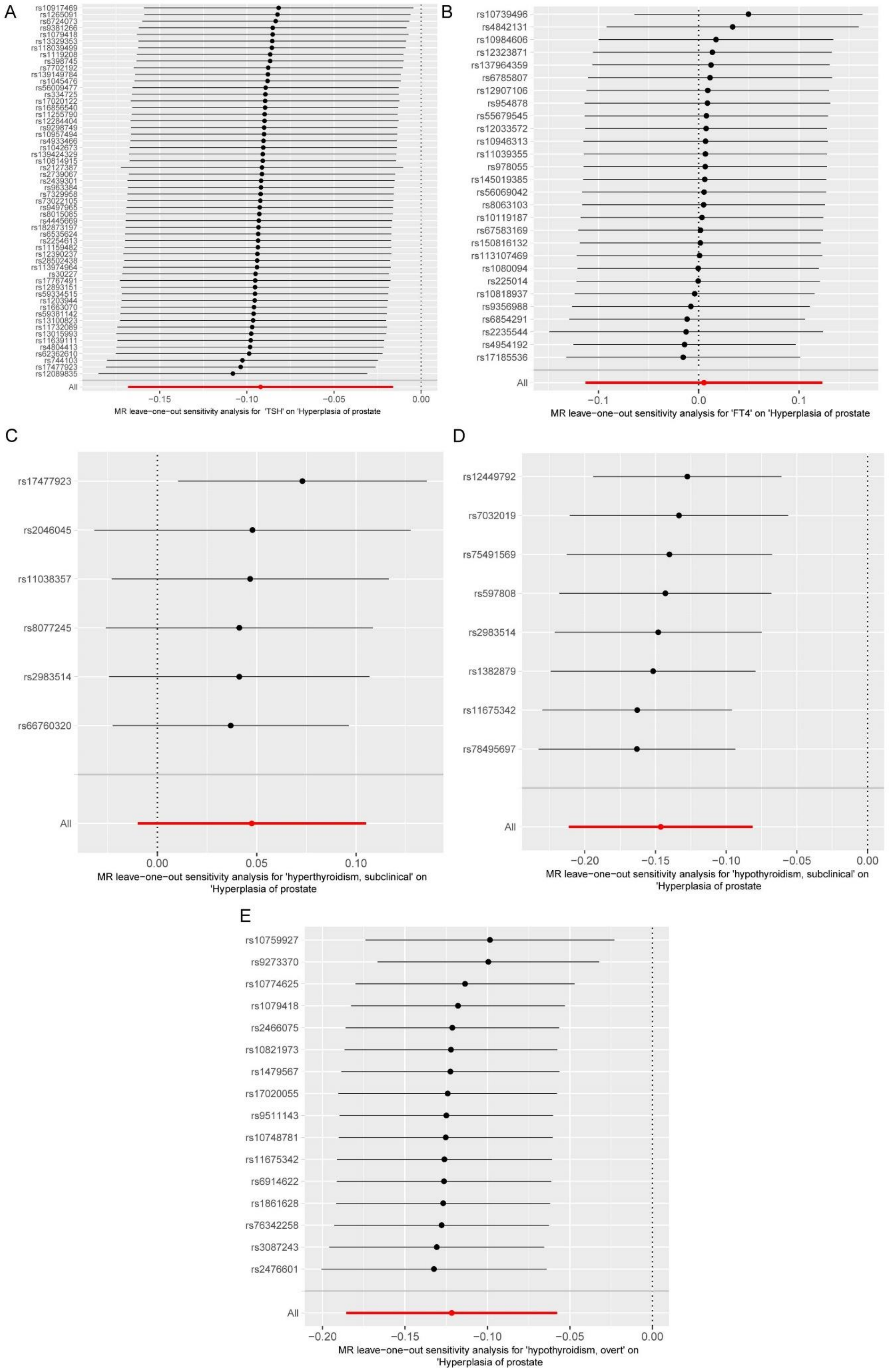
A
All
−0.15
−0.10
−0.05
0.00
MR leave−one−out sensitivity analysis for 'TSH' on 'Hyperplasia of prostate
B
rs10739496
rs4842131
rs10984606
rs12323871
rs137964359
rs6785807
rs12907106
rs954878
rs55679545
rs12033572
rs10946313
rs11039355
rs978055
rs145019385
rs56069042
rs8063103
rs10119187
rs67583169
rs150816132
rs113107469
rs1080094
rs225014
rs10818937
rs9356988
rs6854291
rs2235544
rs4954162
rs17185536
All
−0.1
0.0
0.1
MR leave−one−out sensitivity analysis for 'FT4' on 'Hyperplasia of prostate
C
rs17477923
rs2046045
rs11038357
rs8077245
rs2983514
rs66760320
All
0.00
0.05
0.10
MR leave−one−out sensitivity analysis for 'hyperthyroidism, subclinical' on 'Hyperplasia of prostate
D
rs12449792
rs7032019
rs75491569
rs597808
rs2983514
rs1382879
rs11675342
rs78495697
All
−0.20
−0.15
−0.10
−0.05
0.00
MR leave−one−out sensitivity analysis for 'hypothyroidism, subclinical' on 'Hyperplasia of prostate
E
rs10759927
rs9273370
rs10774625
rs1079418
rs2466075
rs10821973
rs1479567
rs17020055
rs9511143
rs10748781
rs11675342
rs6914622
rs1861628
rs76342258
rs3087243
rs2476601
All
−0.20
−0.15
−0.10
−0.05
0.00
MR leave−one−out sensitivity analysis for 'hypothyroidism, overt' on 'Hyperplasia of prostate

Supplementary Figure 3. Leave-one-out analysis of the causal association between (A) TSH, (B) FT4, (C) hyperthyroidism, subclinical, (D) hypothyroidism, overt, and (E) hypothyroidism, subclinical individuals at risk of BPH. Each SNP was excluded sequentially and the MR estimate effect was recalculated. Visually, the leave-one-out analysis plot illustrated that the results were not driven by any SNP alone. SNP, single-nucleotide polymorphism; MR, Mendelian randomization; BPH, benign prostate hyperplasia.

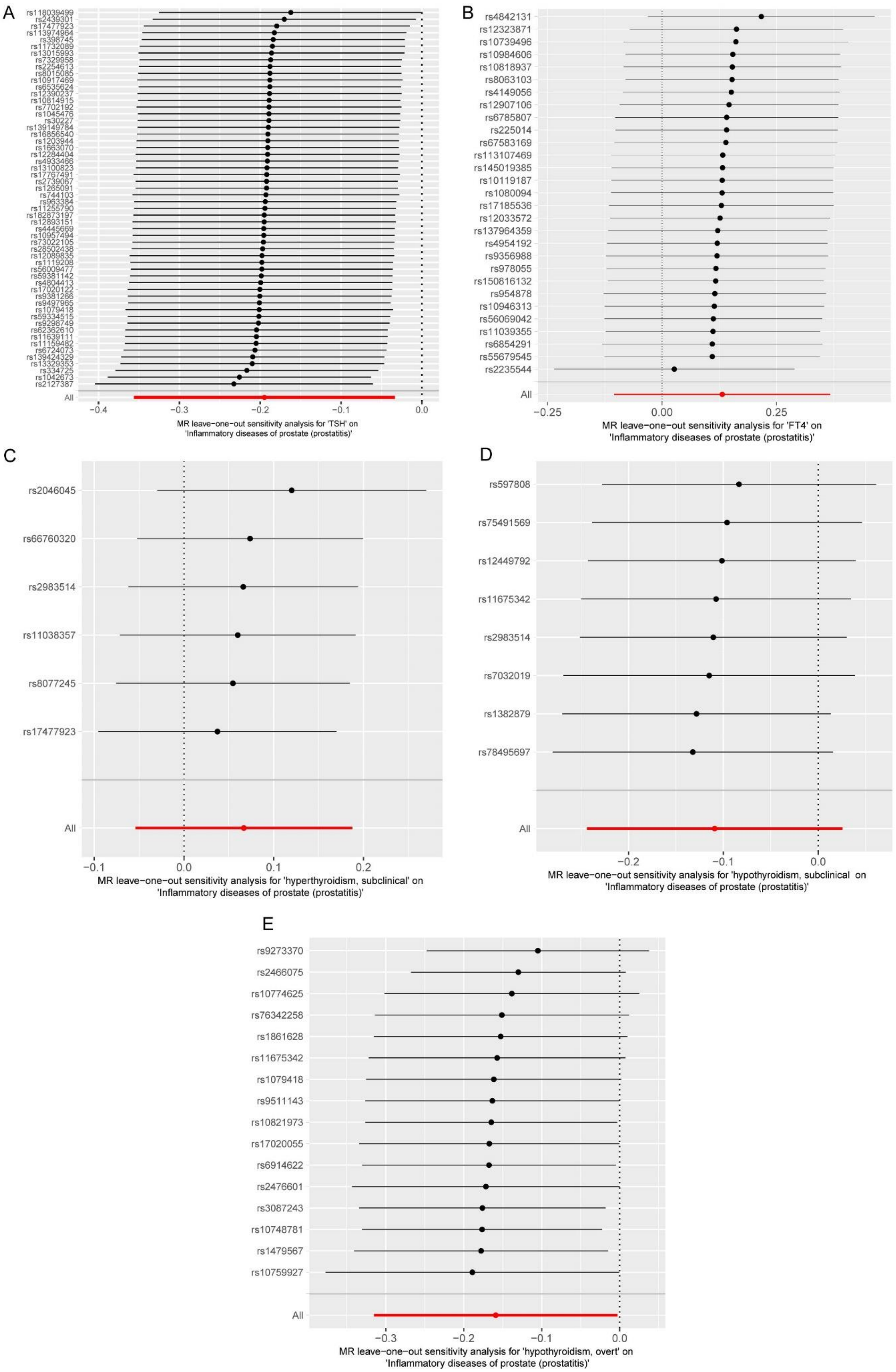
A
All
−0.4
−0.3
−0.2
−0.1
0.0
MR leave−one−out sensitivity analysis for 'TSH' on
'Inflammatory diseases of prostate (prostatitis)'
B
rs4842131
rs12323871
rs10739496
rs10984606
rs10818937
rs8063103
rs4149056
rs12907106
rs6785807
rs225014
rs67583169
rs113107469
rs145019385
rs10119187
rs1080094
rs17185536
rs12033572
rs137964359
rs4954192
rs9356988
rs978055
rs150816132
rs954878
rs10946313
rs56069042
rs11039355
rs6854291
rs55679545
rs2235544
All
−0.25
0.00
0.25
MR leave−one−out sensitivity analysis for 'FT4' on
'Inflammatory diseases of prostate (prostatitis)'
C
rs2046045
rs66760320
rs2983514
rs11038357
rs8077245
rs17477923
All
−0.1
0.0
0.1
0.2
MR leave−one−out sensitivity analysis for 'hyperthyroidism, subclinical' on
'Inflammatory diseases of prostate (prostatitis)'
D
rs597808
rs75491569
rs12449792
rs11675342
rs2983514
rs7032019
rs1382879
rs78495697
All
−0.2
−0.1
0.0
MR leave−one−out sensitivity analysis for 'hypothyroidism, subclinical on
'Inflammatory diseases of prostate (prostatitis)'
E
rs9273370
rs2466075
rs10774625
rs76342258
rs1861628
rs11675342
rs1079418
rs9511143
rs10821973
rs17020055
rs6914622
rs2476601
rs3087243
rs10748781
rs1479567
rs10759927
All
−0.3
−0.2
−0.1
0.0
MR leave−one−out sensitivity analysis for 'hypothyroidism, overt' on
'Inflammatory diseases of prostate (prostatitis)'

Supplementary Figure 4. Leave-one-out analysis of the causal association between (A) TSH, (B) FT4, (C) hyperthyroidism, subclinical, (D) hypothyroidism, overt, and (E) hypothyroidism, subclinical individuals at risk of prostatitis. Each SNP was excluded sequentially and the MR estimate effect was recalculated. Visually, the leave-one-out analysis plot illustrated that the results were not driven by any SNP alone. SNP, single-nucleotide polymorphism; MR, Mendelian randomization.

2 Supplementary Tables

Supplementary Table 1 Data source and study population

**Source**	**Thyroid function exposure**	**GWAS**	**Population**
PMID: 33899528	TSH	ThyroidOmics	54,288<br>TSH within cohort-specific reference range without overt thyroid disease (thyroid surgery or medication use) and analyzed as continuous variable after inverse normal transformation.
PMID: 33899528	FT4	ThyroidOmics	49,269<br>fT4 within cohort-specific reference range without overt thyroid disease (thyroid surgery or medication use) and analyzed as continuous variable after inverse normal transformation
PMID: 33548002	Subclinical hypothyroidism	ThyroidOmics	3,440 cases and 49,983 controls Cases defined on the basis of TSH level above the reference range, but without overt thyroid disease (thyroid surgery or medication use). Thus including primarily mild phenotype.
PMID: 33548002	Overt hypothyroidism	23andMe	8,000 cases and 117,000 controls Cases included subclinical and overt hypothyroidism, including thyroid surgery and medication use (23andMe).
PMID: 33548002	Subclinical hyperthyroidism	ThyroidOmics	1,840 cases and 49,983 controls. Cases defined on the basis of TSH level below the reference range, but without overt thyroid disease (thyroid surgery or medication use). Thus including primarily mild phenotype.
http://r5.finngen.fi/pheno/N14_PROSTHYPERPLA	Hyperplasia of prostate	FinnGen	13,118 cases and 72,799 controls. ICD 2019 code: N41
http://r5.finngen.fi/pheno/N14_PROSTATITIS	Inflammatory diseases of prostate (prostatitis)	FinnGen	1,859 cases and 72,799 controls. ICD 2019 code: N40

Supplementary Table 2 Genetic variants

exposure	gene	snp	effect allele	beta	standard error	*p*	F
TSH	CAPZB	rs12089835	T	0.0725	0.0065	1.27E-28	124.41
TSH	CAPZB	rs10917469	A	0.1112	0.0085	3.95E-39	171.15
TSH	CAPZB	rs74804879	T	0.0501	0.0065	1.22E-14	59.41
TSH	NFIA	rs334725	A	0.1737	0.0147	2.45E-32	139.63
TSH	VAV3	rs17020122	T	0.1044	0.0114	5.32E-20	83.87
TSH	IGFBP5	rs16856540	T	-0.0549	0.0084	7.81E-11	42.72
TSH	IGFBP5	rs13015993	A	0.0818	0.0069	4.52E-32	140.54
TSH	DIRC3	rs6724073	T	0.0508	0.0079	1.35E-10	41.35
TSH	SYN2	rs1663070	T	-0.0463	0.007	3.49E-11	43.75
TSH	TM4SF4	rs28502438	T	0.0338	0.0061	3.70E-08	30.7
TSH	IGF2BP2	rs13100823	T	-0.0406	0.0066	6.76E-10	37.84
TSH	HES1	rs59381142	A	-0.058	0.0076	1.70E-14	58.24
TSH	NR3C2	rs6535624	A	0.0419	0.0062	1.60E-11	45.67
TSH	NR3C2	rs11732089	T	0.115	0.0076	1.73E-51	228.96
TSH	PDE8B	rs62362610	C	0.0726	0.0118	7.73E-10	37.85
TSH	PDE8B	rs1119208	T	0.0457	0.0064	6.65E-13	50.99
TSH	PDE8B	rs139424329	A	-0.2	0.0322	5.14E-10	38.58
TSH	PDE8B	rs2127387	A	0.1435	0.0062	#######	535.7
TSH	PDE8B	rs7702192	A	0.0697	0.0061	2.61E-30	130.56
TSH	PDE8B	rs113974964	T	-0.1237	0.0146	2.06E-17	71.78
TSH	PDE8B	rs139149784	A	0.1556	0.0285	4.97E-08	29.81

TSH	PDE8B	rs182873197	T	-0.0799	0.0142	1.71E-08	31.66
TSH	PSORS1C1	rs1265091	T	0.0571	0.0086	3.20E-11	44.08
TSH	VEGFA/LOC100132354	rs744103	A	0.0919	0.0069	6.73E-41	177.39
TSH	VEGFA/LOC100132354	rs9381266	T	0.0726	0.007	1.84E-25	107.57
TSH	SASH1	rs9497965	T	0.0444	0.0062	9.81E-13	51.28
TSH	PDE10A	rs73022105	T	0.1049	0.0155	1.20E-11	45.8
TSH	PDE10A	rs1079418	A	0.1009	0.0066	8.23E-53	233.72
TSH	SLC25A37	rs56009477	A	0.0524	0.0084	3.72E-10	38.91
TSH	NRG1	rs2439301	A	-0.0587	0.0076	8.15E-15	59.66
TSH	SULF1	rs10957494	A	-0.0402	0.0066	1.10E-09	37.1
TSH	TG	rs118039499	A	0.1837	0.024	1.99E-14	58.59
TSH	TG	rs2739067	A	-0.0415	0.0062	2.43E-11	44.8
TSH	GLIS3	rs10814915	T	0.0421	0.0061	5.06E-12	47.63
TSH	C9orf92	rs9298749	A	-0.0393	0.0064	8.80E-10	37.71
TSH	GATA3	rs11255790	T	-0.041	0.0066	6.83E-10	38.59
TSH	PTEN	rs4933466	A	0.0395	0.0063	5.13E-10	39.31
TSH	NKX2-3	rs200574439	A	-0.0467	0.0064	3.69E-13	53.24
TSH	PRDM11	rs12284404	A	-0.0667	0.0069	2.48E-22	93.44
TSH	CADM1	rs4445669	T	-0.0397	0.0061	5.76E-11	42.36
TSH	SPATA13	rs7329958	T	-0.0439	0.0065	1.13E-11	45.61
TSH	MBIP	rs398745	A	-0.052	0.0062	3.97E-17	70.34
TSH	MBIP	rs2254613	T	-0.0346	0.0063	3.44E-08	30.16
TSH	TSHR	rs11159482	T	0.0846	0.0129	6.30E-11	43.01

TSH	TSHR	rs59334515	T	-0.0539	0.0073	1.10E-13	54.52
TSH	TSHR	rs12893151	A	-0.0624	0.0078	1.02E-15	64
TSH	ITPK1	rs8015085	A	0.0671	0.0077	2.45E-18	75.94
TSH	FAM227B/FGF7	rs17477923	T	0.0826	0.0069	2.57E-33	143.31
TSH	FAM227B/FGF7	rs11639111	T	0.045	0.0062	3.60E-13	52.68
TSH	DET1	rs13329353	T	0.0614	0.0065	5.17E-21	89.23
TSH	ADCY9	rs1045476	A	0.049	0.0082	2.36E-09	35.71
TSH	MIR365A	rs30227	T	-0.0468	0.0063	7.59E-14	55.18
TSH	MAF	rs17767491	A	0.0883	0.0065	3.35E-42	184.54
TSH	NSF	rs77819282	A	0.0452	0.0074	1.13E-09	37.31
TSH	SOX9	rs1042673	A	-0.0546	0.0061	3.57E-19	80.12
TSH	SOX9	rs963384	T	0.0351	0.0063	2.77E-08	31.04
TSH	INSR	rs4804413	T	0.0532	0.0062	8.64E-18	73.63
TSH	FOXA2	rs1203944	T	-0.0509	0.0073	2.42E-12	48.62
TSH	PRKX	rs12390237	A	-0.0458	0.0068	1.74E-11	45.36
FT4	DIO1	rs145019385	T	0.1813	0.0317	1.13E-08	32.71
FT4	DIO1	rs12033572	C	0.1152	0.0171	1.45E-11	45.39
FT4	DIO1	rs2235544	A	0.1387	0.0065	#######	455.33
FT4	DIO1	rs954878	A	-0.0582	0.0065	4.83E-19	80.17
FT4	ACMSD	rs4954192	T	-0.0409	0.0071	8.38E-09	33.18
FT4	SOX2-OT	rs6785807	A	-0.059	0.0093	2.47E-10	40.25
FT4	AADAT	rs6854291	A	0.117	0.0114	1.35E-24	105.33
FT4	ID4	rs10946313	T	0.0455	0.0068	2.28E-11	44.77
FT4	SLC17A4	rs9356988	A	-0.051	0.0073	3.56E-	48.81

						12	
FT4	SLC17A4	rs137964359	T	-0.1999	0.0315	2.13E-10	40.27
FT4	LOC728012	rs17185536	T	0.0726	0.0081	1.93E-19	80.33
FT4	CA8	rs67583169	C	0.0613	0.0095	9.99E-11	41.64
FT4	GLIS3	rs10119187	T	0.0497	0.0085	4.11E-09	34.19
FT4	FOXE1	rs10739496	T	0.0777	0.0068	4.20E-30	130.56
FT4	FOXE1	rs10984606	T	-0.0398	0.0065	1.17E-09	37.49
FT4	NEK6	rs10818937	T	-0.0475	0.007	1.31E-11	46.05
FT4	LHX3	rs4842131	T	-0.1037	0.0075	7.68E-44	191.18
FT4	LHX3	rs55679545	A	0.044	0.0076	8.42E-09	33.52
FT4	FNBP4	rs11039355	T	-0.0385	0.007	3.51E-08	30.25
FT4	SLCO1B1	rs4149056	T	-0.0506	0.0089	1.34E-08	32.32
FT4	DIO2	rs150816132	A	-0.2197	0.0398	3.47E-08	30.47
FT4	DIO2	rs978055	A	0.0384	0.0067	1.06E-08	32.85
FT4	DIO2	rs225014	T	0.0535	0.0067	1.83E-15	63.76
FT4	DIO3OS	rs12323871	T	-0.0474	0.0084	1.42E-08	31.84
FT4	DIO3OS	rs11626434	C	0.0583	0.0069	4.08E-17	71.39
FT4	USP3	rs12907106	C	-0.0407	0.0074	3.67E-08	30.25
FT4	SNX29	rs8063103	C	-0.0522	0.0092	1.61E-08	32.19
FT4	NCOR1	rs11078333	A	0.0513	0.0072	9.91E-13	50.77
FT4	SLC25A52	rs1080094	A	-0.0415	0.0066	4.06E-10	39.54
FT4	SLC25A52	rs113107469	T	0.1996	0.022	1.00E-19	82.31
FT4	MC4R	rs56069042	A	0.1061	0.0186	1.16E-08	32.54
hypothyroidis	CAPZB	rs7549156	C	0.238	0.037	8.7E-	41

m, subclinical		9				08	
hypothyroidism, subclinical	VAV3	rs78495697	T	0.316	0.041	2.6E-11	59
hypothyroidism, subclinical	TPO	rs11675342	T	0.16	0.025	1.5E-07	41
hypothyroidism, subclinical	PDE8B	rs1382879	C	0.153	0.026	2.1E-06	35
hypothyroidism, subclinical	PDE10A	rs2983514	A	0.154	0.027	0.000016	33
hypothyroidism, subclinical	FOXE1	rs7032019	A	0.248	0.027	5.55E-17	84
hypothyroidism, subclinical	ATXN2	rs597808	A	0.18	0.026	3.5E-09	48
hypothyroidism, subclinical	FMNL1	rs12449792	T	0.157	0.028	0.000024	31
hypothyroidism, overt	CAPZB	rs12138950	A	0.102033	0.017795507	7.5E-09	33
hypothyroidism, overt	VAV3	rs17020055	C	0.201	0.021	2.7E-22	92
hypothyroidism, overt	PTPN22	rs2476601	A	0.241	0.02	2.1E-31	145
hypothyroidism, overt	TPO	rs11675342	T	0.0944	0.013	6.4E-14	53
hypothyroidism, overt	IGFBP5	rs1861628	G	0.083422	0.014313188	5.6E-09	34
hypothyroidism, overt	CTLA4	rs3087243	G	0.099845	0.012457957	1.4E-15	64
hypothyroidism, overt	NR3C2	rs76342258	G	0.139	0.0164	8.1E-18	72
hypothyroidism, overt	PDE8B	rs1479567	A	0.127833	0.012460841	1.4E-23	105
hypothyroidism, overt	VEGFA	rs10223666	C	0.087	0.014	4.6E-10	39
hypothyroidism, overt	PDE10A	rs1079418	A	0.092115	0.013702175	9.3E-12	45
hypothyroidism, overt	SASH1	rs6914622	T	0.103459	0.013332935	1.2E-14	60
hypothyroidism, overt	HLA	rs9273370	A	0.160169	0.013177455	4.5E-34	148
hypothyroidism, overt	NRG1	rs2466075	G	0.081	0.013	5.9E-10	39
hypothyroidism, overt	FOXE1	rs10759927	G	0.272	0.014	9.7E-90	377
hypothyroidism, overt	NKX2-3	rs10748781	C	0.084341	0.013133393	1.4E-10	41
hypothyroidism, overt	ZNF365	rs10821973	G	0.067659	0.012841024	4.1E-08	28

hypothyroidism, overt	ATXN2	rs10774625	A	0.134	0.0125	1.4E-26	115
hypothyroidism, overt	SPATA13	rs9511143	T	0.083382	0.0166429 9	2.6E-08	25
hyperthyroidism, subclinical	CAPZB	rs12138950	C	0.258	0.042	5.5E-10	37.735
hyperthyroidism, subclinical	PDE8B	rs2046045	T	0.329	0.034	4.4E-22	93.634
hyperthyroidism, subclinical	PDE10A	rs2983514	G	0.199	0.032	5.2E-10	38.673
hyperthyroidism, subclinical	VEGFA	rs66760320	T	0.192	0.035	4.4E-08	30.093
hyperthyroidism, subclinical	PRDM11	rs11038357	A	0.235	0.035	3.1E-11	45.082
hyperthyroidism, subclinical	FGF7	rs17477923	C	0.245	0.034	6.1E-13	51.925
hyperthyroidism, subclinical	SOX9	rs8077245	T	0.205	0.033	5E-10	38.59

Supplementary Table 3 Five methods to assess the causal role of thyroid function in the risk of benign prostate hyperplasia and prostatitis

	benign prostate hyperplasia			prostatitis		
Method	SNPs	OR (95% CI)	*p* value	SNPs	OR (95% CI)	*p* value
TSH						
IVW	56	0.912(0.845-0.984)	1.76E-02	56	0.823(0.700-0.967)	1.80E-02
MR Egger	56	0.887(0.732-1.075)	2.28E-01	56	0.908(0.604-1.365)	6.45E-01

Weighted median	56	0.908(0.802-1.026)	1.22E-01	56	0.800(0.631-1.015)	6.63E-02
Weighted mode	56	0.926(0.774-1.109)	4.07E-01	56	0.812(0.571-1.153)	2.49E-01
Simple mode	56	0.949(0.748-1.205)	6.71E-01	56	0.633(0.396-1.013)	6.17E-02
FT4						
IVW	28	1.005(0.893-1.131)	9.30E-01	29	1.141(0.901-1.444)	2.75E-01
MR Egger	28	0.976(0.741-1.284)	8.61E-01	29	1.455(0.845-2.504)	1.87E-01
Weighted median	28	1.041(0.871-1.245)	6.57E-01	29	1.185(0.835-1.683)	3.41E-01
Weighted mode	28	1.021(0.836-1.248)	8.38E-01	29	1.444(0.848-2.459)	1.87E-01
Simple mode	28	0.952(0.684-1.324)	7.72E-01	29	1.555(0.732-3.303)	2.60E-01
hypothyroidism, subclinical						
IVW	8	0.864(0.810-0.922)	1.04E-05	8	0.897(0.784-1.026)	1.12E-01
MR Egger	8	0.886(0.668-1.176)	4.34E-01	8	0.968(0.562-1.668)	9.11E-01
Weighted median	8	0.857(0.786-0.934)	4.61E-04	8	0.912(0.772-1.078)	2.80E-01

Weighted mode	8	0.842(0.740-0.959)	3.57E-02	8	0.906(0.717-1.146)	4.38E-01
Simple mode	8	0.853(0.743-0.979)	5.87E-02	8	0.881(0.689-1.126)	3.45E-01
hypothyroidism, overt						
IVW	16	0.885(0.831-0.944)	1.92E-04	16	0.853(0.730-0.997)	4.61E-02
MR Egger	16	0.805(0.692-0.937)	1.44E-02	16	0.875(0.596-1.286)	5.08E-01
Weighted median	16	0.882(0.804-0.967)	7.26E-03	16	0.926(0.761-1.126)	4.41E-01
Weighted mode	16	0.850(0.756-0.955)	1.57E-02	16	0.938(0.754-1.166)	5.71E-01
Simple mode	16	0.997(0.833-1.193)	9.72E-01	16	0.982(0.706-1.366)	9.15E-01
hyperthyroidism, subclinical						
IVW	6	1.049(0.990-1.111)	1.05E-01	6	1.069(0.947-1.206)	2.79E-01
MR Egger	6	0.924(0.685-1.246)	6.32E-01	6	0.845(0.459-1.556)	6.18E-01
Weighted median	6	1.051(0.975-1.133)	1.92E-01	6	1.078(0.924-1.256)	3.40E-01

Weighted mode	6	1.055(0.965-1.153)	2.90E-01	6	0.986(0.806-1.206)	8.97E-01
Simple mode	6	1.071(0.958-1.196)	2.83E-01	6	1.109(0.891-1.382)	3.97E-01
